# Inhibition of lymphatic proliferation by the selective VEGFR-3 inhibitor SAR131675 ameliorates diabetic nephropathy in *db/db* mice

**DOI:** 10.1038/s41419-019-1436-1

**Published:** 2019-03-04

**Authors:** Seun Deuk Hwang, Joon Ho Song, Yaeni Kim, Ji Hee Lim, Min Young Kim, Eun Nim Kim, Yu Ah Hong, Sungjin Chung, Bum Soon Choi, Yong-Soo Kim, Cheol Whee Park

**Affiliations:** 1 Department of Internal Medicine, Division of Nephrology, College of Medicine, The Inha University, Incheon, Korea; 2 Department of Internal Medicine, Division of Nephrology, College of Medicine, The Catholic University of Korea, Seoul, Korea

## Abstract

Recent studies have demonstrated that chronic inflammation-induced lymphangiogenesis plays a crucial role in the progression of various renal diseases, including diabetic nephropathy. SAR131675 is a selective vascular endothelial cell growth factor receptor-3 (VEGFR-3)-tyrosine kinase inhibitor that acts as a ligand for VEGF-C and VEGF-D to inhibit lymphangiogenesis. In this study, we evaluated the effect of SAR131675 on renal lymphangiogenesis in a mouse model of type 2 diabetes. Male C57BLKS/J *db/m* and *db/db* mice were fed either a regular chow diet or a diet containing SAR131675 for 12 weeks from 8 weeks of age. In addition, we studied palmitate-induced lymphangiogenesis in human kidney-2 (HK2) cells and RAW264.7 monocytes/macrophages, which play a major role in lymphangiogenesis in the kidneys. SAR131475 ameliorated dyslipidemia, albuminuria, and lipid accumulation in the kidneys of *db/db* mice, with no significant changes in glucose and creatinine levels and body weight. Diabetes-induced systemic inflammation as evidenced by increased systemic monocyte chemoattractant protein-1 and tumor necrosis factor-α level was decreased by SAR131475. SAR131475 ameliorated the accumulation of triglycerides and free fatty acids and reduced inflammation in relation to decreased chemokine expression and pro-inflammatory M1 macrophage infiltration in the kidneys. Downregulation of VEGF-C and VEGFR-3 by SAR131475 inhibited lymphatic growth as demonstrated by decreased expression of LYVE-1 and podoplanin that was further accompanied by reduced tubulointerstitial fibrosis, and inflammation in relation to improvement in oxidative stress and apoptosis. Treatment with SAR131475 improved palmitate-induced increase in the expression of VEGF-C, VEGFR-3, and LYVE-1, along with improvement in cytosolic and mitochondrial oxidative stress in RAW264.7 and HK2 cells. Moreover, the enhanced expression of M1 phenotypes in RAW264.7 cells under palmitate stress was reduced by SAR131475 treatment. The results suggest that modulation of lymphatic proliferation in the kidneys is a new treatment approach for type 2 diabetic nephropathy and that SAR131675 is a promising therapy to ameliorate renal damage by reducing lipotoxicity-induced lymphangiogenesis.

## Introduction

Diabetic nephropathy is a leading cause of end-stage renal disease worldwide, including Korea^1^. Hyperglycemia-induced oxidative stress and inflammation play major roles in the development and progression of diabetic chronic kidney disease. Furthermore, lipid accumulation is a pathological feature of every type of kidney injury^2^. Recent studies have demonstrated that ectopic accumulation of free fatty acids and triglycerides in the kidneys also plays a crucial role in the progression of diabetic renal damage^3,4^, suggesting that lipotoxicity-induced oxidative stress and inflammation may critically contribute to the pathogenesis of diabetic chronic kidney disease.

It is well known that functional lymphatics normally clear fluid, macromolecules, and immune cells both passively and actively from the peripheral interstitium. However, disorganized lymphatic expansion leads to failure of immune cell clearance, and, consequently, to chronic inflammation^5^. Lymphangiogenesis is often observed during the development of tissue fibrosis^6^. In a recent study, proteinuria triggered renal lymphangiogenesis and, subsequently, tubulointerstitial fibrosis, which were associated with macrophage activation in the fibrotic interstitium by autocrine and paracrine actions through the production of cytokines such as interleukin-1^7^. In addition, in a unilateral ureteral obstruction-induced rat model, macrophages, especially M2-polarized macrophages, and proximal tubule cells, upregulated vascular endothelial cell growth factor-C (VEGF-C) expression via tumor nuclear factor-α (TNF-α) and transforming growth factor (TGF)-β, leading to lymphangiogenesis by activation of VEGF receptor-3 (VEGFR-3) on lymphatic endothelial cells^8^. The activation of VEGFR-3 by its ligands VEGF-C and VEGF-D is the key signaling mechanism for lymphangiogenesis^9^. Serum levels of VEGF-C and VEGF-D are elevated in inflammatory disease^10,11^, and classically activated macrophages, known as the M1 phenotype, are stimulated by interferon-γ and TNF-α, and provoke the secretion of cytotoxic agents, such as nitric oxide and pro-inflammatory cytokines, including interleukin-1 (IL-1), IL-6, IL-12, IL-23, and TNF-α. TNF-α increases VEGF-C expression in proximal tubular cells, at which point VEGF-C protein is expressed in M1-polarized macrophages^12^. However, the role of polarized macrophages in lymphangiogenesis is not well defined and is fiercely debated^8^.

SAR131675 is a selective VEGFR-3-tyrosine kinase (TK) inhibitor that is 10-fold and 50-fold more selective for VEGFR-3-TK than VEGFR-1/VEGFR-2, and it is known to have anti-lymphangiogenic, anti-tumoral, and anti-metastatic activities^13^. This potent and selective VEGFR-3 inhibitor inhibits the activation of VEGFR-3-TK and, consequently, the autophosphorylation of VEGFR-3. Further, it acts as a ligand for VEGF-C and VEGF-D, which are involved in lymphangiogenesis. Therefore, blocking VEGF-C, VEGF-D, or VEGFR-3 might inhibit lymphangiogenesis^14^.

In the current study, we hypothesized that SAR131675 can prevent diabetic nephropathy by inhibiting renal lymphangiogenesis, and that it may be mediated through macrophage polarization in the proximal epithelial tubular cells under palmitate-induced lipotoxic condition.

## Materials and methods

### Experimental methods

Six-week-old male C57BLKS/J *db/m* and *db/db* mice, purchased from Jackson Laboratories (Bar Harbor, ME, USA), received either a regular diet of chow or a diet containing SAR131675 (Selleckchem) (30 mg/kg) for 12 weeks starting at 8 weeks of age (*n* = 8 in each of the four groups). At week 20, all animals were anesthetized by intraperitoneal injection of 30 mg/kg tiletamine plus zolazepam (Zoletil; Virbac, Carros, France) and 10 mg/kg xylazine hydrochloride (Rompun; Bayer, Leuverkusen, Germany). Blood was collected from the left ventricle and the plasma was stored at –70 °C for subsequent analyses.

### Ethics statement

All animal experiments were performed in accordance with the Laboratory Animals Welfare Act and the Guide for the Care and Use of Laboratory Animals (National Institutes of Health Publication No. 85-23, revised 1996), and were approved by the Institutional Animal Care and Use Committee (IACUC) at the College of Medicine, the Catholic University of Korea (CUMC-2015-0170-02). All procedures complied with the Guide for the Care and Use of Laboratory Animals (National Institutes of Health Publication No. 85-23, revised 1996).

### Measurement of serum parameters

After 12 weeks of SAR131675 treatment, blood glucose was measured using an Accu-check meter (Roche Diagnostics, St. Louis, MO, USA). Hemoglobin A1c (HbA1c) was determined from red cell lysates by high-performance liquid chromatography (HPLC) (Bio-Rad, Richmond, CA, USA). Total cholesterol, triacylglycerol, and nonesterified free fatty acid concentrations were measured with an autoanalyzer (Hitachi 917; Hitachi, Tokyo, Japan) using commercial kits (Wako, Osaka, Japan).

### Assessment of renal function, oxidative stress, and intrarenal lipids

Twenty-four-hour urine samples were obtained from the mice at 20 weeks of age using metabolic cages, and urinary albumin concentrations were measured by immunoassay (Bayer, Elkhart, IN, USA). Plasma and urine creatinine concentrations were measured using HPLC (Beckman Instruments, Fullerton, CA, USA). To evaluate oxidative stress, we measured the 24-h urinary 8-hydroxy-2′-deoxyguanosine (8-OH-dG; Oxis Health Products, Portland, OR, USA) and 8-epi-prostaglandin F2α (Oxis Research, Foster City, CA, USA) levels. Kidney lipids were extracted using the method of Bligh and Dyer with slight modifications as previously described (Waco, Osaka, Japan)^15^. To evaluate the effect of SAR131675 on lipid accumulation in the glomerulus, we performed oil red O staining of frozen renal tissue.

### Enzyme-linked immunosorbent assay to assess inflammation markers

To evaluate inflammatory markers, serum samples were analyzed using a mouse Magnetic Luminex Screening assay containing a premixed multi-analyte kit for murine TNF-α and monocyte chemoattractant protein-1 (MCP-1) (R&D Systems, Minneapolis, MN, USA) on a Luminex 200 (Austin, TX, USA) according to the manufacturer’s instructions. Secretory level of MCP-1 in kidney tissue lysate was determined by a commercial ELISA kit purchased from Abcam Inc. (Cambridge, UK). The color generated was determined by measuring the optical density value at 450 nm with a spectrophotometric microtiter plate reader (Molecular Devices Corp., Sunnyvale, CA, USA).

### Light microscopy

Histology was assessed following periodic acid-Schiff (PAS) and Masson’s trichrome staining. The mesangial matrix and glomerular tuft areas were quantified for each glomerular cross-section using PAS-stained sections as previously reported^16^. More than 30 glomeruli, cut through the vascular pole, were counted per kidney and the average was used for analysis. A finding of tubulointerstitial fibrosis was defined as a matrix-rich expansion of the interstitium in Masson’s trichrome-stained sections.

### Immunohistochemistry and TUNEL assay

For immunohistochemistry, 4-μm sections were deparaffinized, hydrated in ethanol, treated with an antigen-unmasking solution of 10 mmol/L sodium citrate buffers, pH 6.0, and washed with phosphate-buffered saline (PBS). The sections were incubated with 3% H_2_O_2_ in methanol to block endogenous peroxidase activity. Nonspecific binding was blocked with 10% normal goat serum in PBS. The sections were incubated overnight with antibodies against TGF-β1 (1:100; R&D Systems, Minneapolis, MN, USA), type IV collagen (Col IV) (1:200; Biodesign International, Saco, ME, USA), F4/80 (1:50; Serotec, Oxford, UK), VEGF-C (Invitrogen, Carlsbad, CA, USA), LYVE-1 (Novus Biologicals, Littleton, CO, USA), podoplanin (ReliaTech GmbH, Wolfenbüttel, Germany), and α-smooth muscle actin (α-SMA) (Abcam, Cambridge, UK) in a humidified chamber at 4 °C. Antibody binding was visualized with peroxidase-conjugated secondary antibody using Vector Impress kits (Vector Laboratories, Burlingame, CA, USA) and 3, 3-diaminobenzidine substrate solution. The sections were dehydrated in ethanol, cleared in xylene, and mounted without counterstaining. All sections were examined in a blinded manner by light microscopy (Olympus BX-50; Olympus Optical, Tokyo, Japan). For the quantification of proportional areas of staining, ~20 views (magnification) were used. These areas were randomly located in the renal cortex and the corticomedullary junction of each slide (Scion Image Beta 4.0.2, Frederik, MD, USA).The proportion of apoptotic cells was determined using ApopTaqIn Situ Apoptosis Detection kits (Chemicon-Millipore, Billerica, MA, USA), based on the terminal deoxynucleotidyl transferase-mediated dUTP nick-end labeling (TUNEL) assay.

### Western blot analysis

Protein extracts were prepared from cultured cells and homogenates of mouse renal cortex or medulla by using mammalian protein extraction reagent (Intron Biotechnology, Gyeonggi-Do, Korea) lysis buffer to which protease inhibitors (Roche, Indianapolis) was added, and Western blots were performed with specific antibodies for VEGF-C (Invitrogen, Carlsbad, CA, USA), VEGFR-1 (Epitomics, Burlingame, CA, USA), VEGFR-2 (Cell Signaling Technology, Danvers, MA, USA) and VEGFR3 (Santa Cruz Biotechnology, Santa Cruz, CA, USA), LYVE-1 (Novus Biologicals, Littleton, CO, USA), podoplanin (ReliaTech GmbH, Wolfenbüttel, Germany), α-SMA (Abcam, Cambridge, UK), fibronectin (ProteinTech, Chicago, IL, USA), inducible nitric oxide synthase (iNOS) (BD Biosciences, San Diego, CA, USA), CD68 (Bio-Rad, Richmond, CA, USA), arginase I (Santa Cruz Biotechnology, Santa Cruz, CA, USA), arginase II (Santa Cruz Biotechnology, Santa Cruz, CA, USA), B cell leukemia/lymphoma 2 (Bcl-2), Bcl-2-associated X protein (Bax) (Santa Cruz Biotechnology, Santa Cruz, CA, USA), and β-actin (Sigma-Aldrich, St. Louis, MO, USA). After incubation with horseradish peroxidase-conjugated anti-mouse or anti-rabbit IgG (secondary antibody) (Cell Signaling Technology, Danvers, MA, USA), target proteins were visualized by an enhanced chemiluminescence substrate (ECL Plus; GE Healthcare Bioscience, Piscataway, NJ, USA) and detected by a Vilber chemiluminescence analyzer (Fusion SL 4; Vilber Lourmat, Marne-la-Vallée, France). The density of each band was quantified with the Quantity One software (Bio-Rad Laboratory, Hercules, CA, USA).

### Cell culture

Human kidney-2 cells (HK2) and RAW264.7 mouse monocyte/macrophage cells (ATCC, Manassas, VA, USA) were maintained in keratinocyte-SFM media (Gibco, Grand Island, NY, USA) with supplement and Dulbecco’s modified Eagle's medium (ATCC, Manassas, VA, USA) with 25 mM glucose, respectively, supplemented with 10% fetal bovine serum (FBS) (Hyclone, Logan, UT, USA). HK2 and RAW264.7 cells were then exposed to high-glucose (30 mM d-glucose) and palmitate (500 μM) media with or without an additional 6-h application of SAR131675 (1, 10, and 100 nM).

### Determination of ROS

Reactive oxygen species (ROS) were measured by fluorescence microscopy following treatment of cells with 2 μM dihydroethidium (DHE; Molecular Probes/Invitrogen, Carlsbad, CA, USA) and 5 μM MitoSOX (Thermo Fisher Scientific, Waltham, MA, USA). The fluorescent images were examined under a laser scanning confocal microscope system (Carl Zeiss LSM 700, Oberkochen, Germany).

### Statistical analysis

Data are expressed as the mean ± standard deviation (SD). Differences between groups were examined for statistical significance using analysis of variance with Bonferroni’s correction using SPSS version 11.5 (SPSS, Chicago, IL, USA). A *P* value <0.05 was considered significant.

## Results

### Physical and biochemical characteristics of *db/db* mice treated with SAR131675

**Fig. 1:**
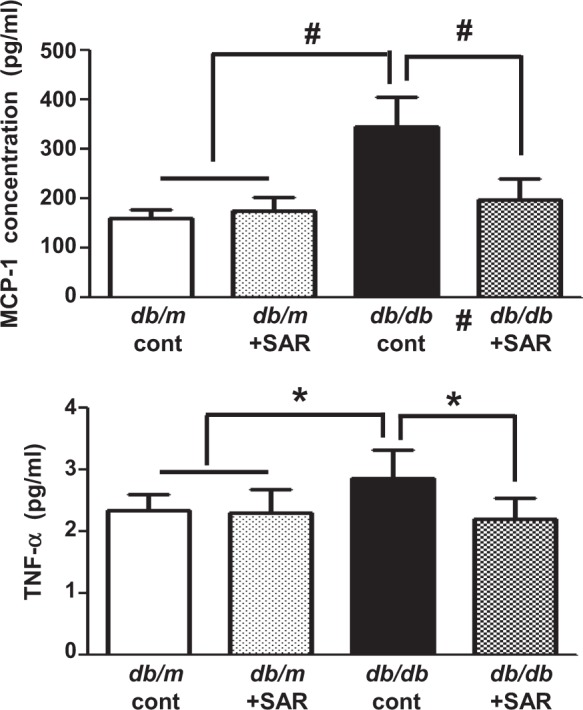
SAR131675 treatment decreases circulating monocyte chemoattractant protein-1 (MCP-1) and tumor necrosis factor-α (TNF-α) level in *db/db* mice. Circulating MCP-1 and TNF-α level were determined at 20 weeks in *db/m* and *db/db* mice treated with or without SAR131675. *P *<* 0.05 and ^#^*P* *<* 0.001 vs. other groups

Body weight, fasting blood glucose, and HbA1c were significantly higher in *db/db* mice than in *db/m* mice, regardless of treatment with SAR131675 (Table 1). Serum creatinine and blood urea nitrogen concentrations were not different among all study groups. SAR131675 treatment significantly decreased serum cholesterol, free fatty acid, and triglyceride levels and albuminuria in *db/db* mice (Table 1) (*P* < 0.05 compared with the *db/db* control group, *P* *<* 0.01 compared with the *db/db* control group, and *P* *<* 0.001 compared with the *db/db* control group and the other groups). Interestingly, SAR131675 treatment significantly lowered diabetes-induced systemic inflammation as indicated by decreased circulating MCP-1 and TNF-α levels in *db/db* mice (Fig. 1).

**Table 1:** Biochemical and physical characteristics of the four groups at the end of the 16-week experimental period

	*dm* cont	*dm* + SAR	*db* cont	*db* + SAR
Body weight (g)	27 ± 5	31 ± 2	41 ± 4^#^	43 ± 7^#^
Blood glucose (mg/dl)	124 ± 47	150 ± 32	596 ± 93^#^	555 ± 55^#^
HbA1c (%)	3.3 ± 0.7	3.2 ± 0.2	11.2 ± 1.8^#^	10.3 ± 2.0^#^
BUN (mg/dl)	21 ± 6	26 ± 8	31 ± 8	28 ± 7
Cr (mg/dl)	0.20 ± 0.01	0.20 ± 0.01	0.19 ± 0.02	0.21 ± 0.03
TC (mg/dl)	81 ± 8	79 ± 5	106 ± 19*	86 ± 10*
FFA (mEg/l)	0.36 ± 0.04	0.45 ± 0.10	1.41 ± 0.37^#^	1.05 ± 0.14^#^
TG (mg/dl)	65 ± 4	64 ± 18	155 ± 60*	109 ± 30*
Urine albumin/Cr (μg/mg)	9 ± 3	12 ± 4	117 ± 11^#^	65 ± 13*

*HbA1c* hemoglobin A1c, *BUN* blood urea nitrogen, *Ca* total calcium, *Cr* creatinine, *FFA* free fatty acid, *TC* total cholesterol, *TG* triglycerides

^*^P  0.05, ^#^*P  *0.001 vs. other groups

### Effects of SAR131675 treatment on renal lipid accumulation and fat concentration

**Fig. 2:**
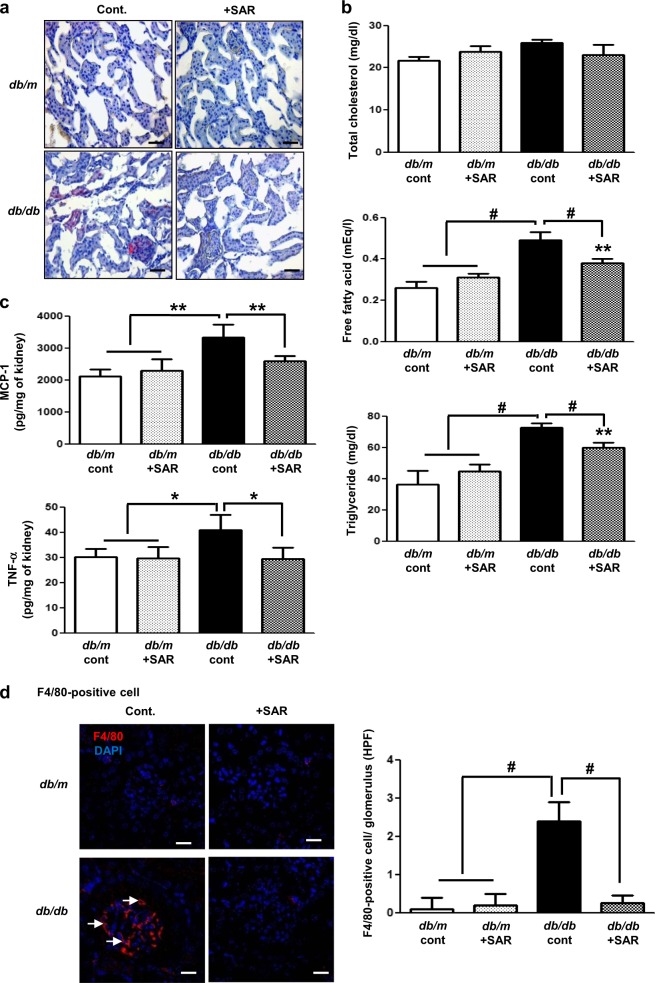

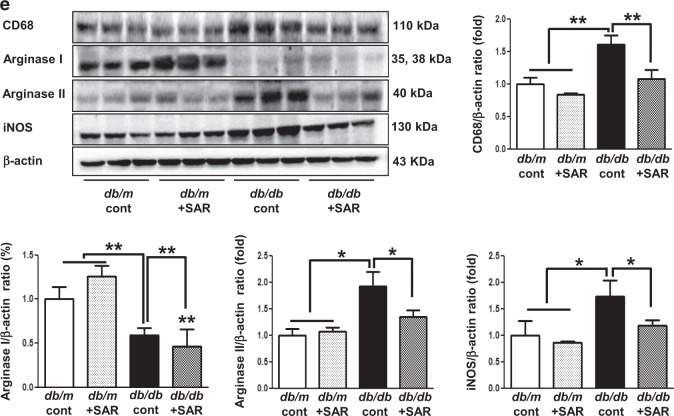
SAR131675 attenuates intrarenal lipid accumulation, inflammation, and macrophage M1 polarization in *db/db* mice. Lipid accumulation in the kidneys, inflammatory cell infiltration, and macrophage polarization were determined at 20 weeks in *db/m* and *db/db* mice treated with or without SAR131675. **a** Representative images of oil red O staining. **b** Intrarenal total cholesterol, free fatty acids, and triglycerides. **c** Monocyte chemoattractant protein-1 (MCP-1) and tumor necrosis factor-α (TNF-α) levels. **d** Representative images of immunohistochemical staining for F4/80-positive cells. **e** Representative western blots for CD68, arginase I, arginase II, inducible nitric oxide synthase (iNOS), and β-actin and quantitative data. *P *<* 0.05, **P *<* 0.01, and ^#^*P* *<* 0.001 vs. other groups

*Db/db* mice showed an increase in lipid accumulation as indicated by oil red O staining and a marked increase in free fatty acid and triglyceride concentrations in the kidneys compared to non-diabetic *db/m* mice (Fig. 2a, b). In *db/db* mice, SAR131675 treatment reduced lipid accumulation as well as free fatty acid and triglyceride deposition (Fig. 2a, b) (*P < 0.05 compared with *db/db* control group and *P* < 0.001 compared with other groups).

### Effects of SAR131675 treatment on the inflammatory response in the kidneys

MCP-1 and TNF-α expression in the kidneys was significantly increased in the *db/db* control group, indicating an enhanced inflammatory response (Fig. 2c). F4/80-positive cell infiltration was significantly increased in the *db/db* control group as well (Fig. 2d). Increased CD68, arginase II, and iNOS activities and decreased arginase I activity, indicating M1 macrophage activation, were reversed by SAR131675 treatment in *db/db* mice (Fig. 2e).

### Effects of SAR131675 treatment on VEGF-C, VEGFR-1, VEGFR-2, and VEGFR-3 expression in the renal cortex and medulla

**Fig. 3:**
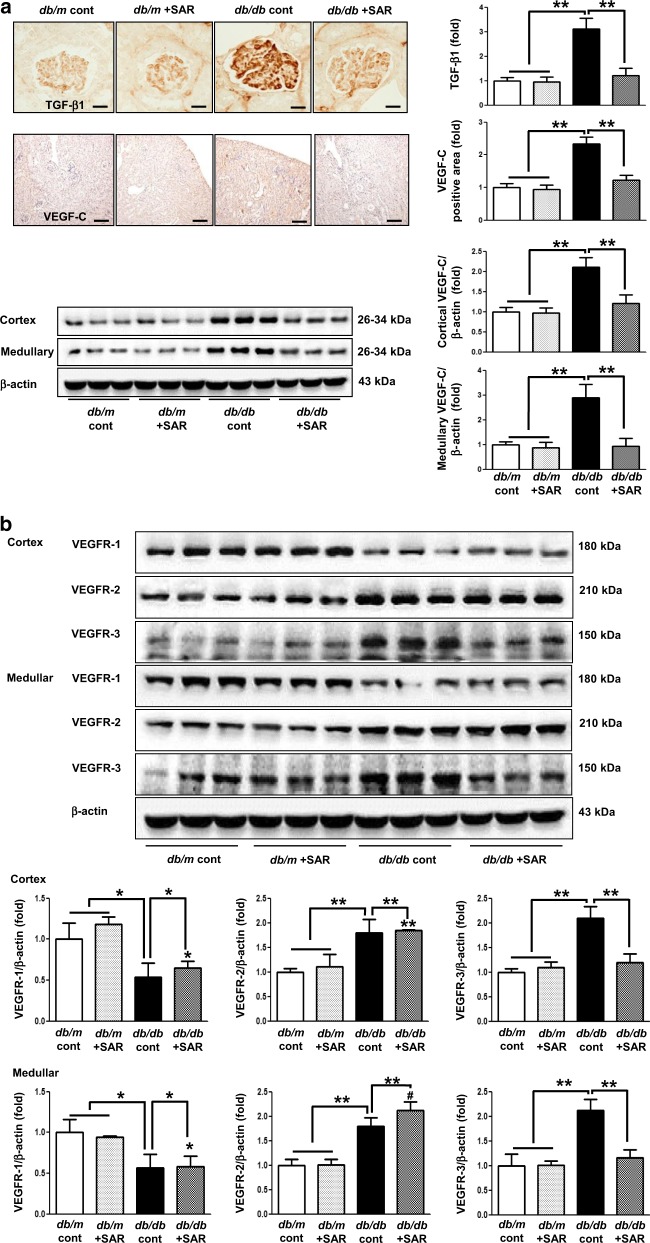

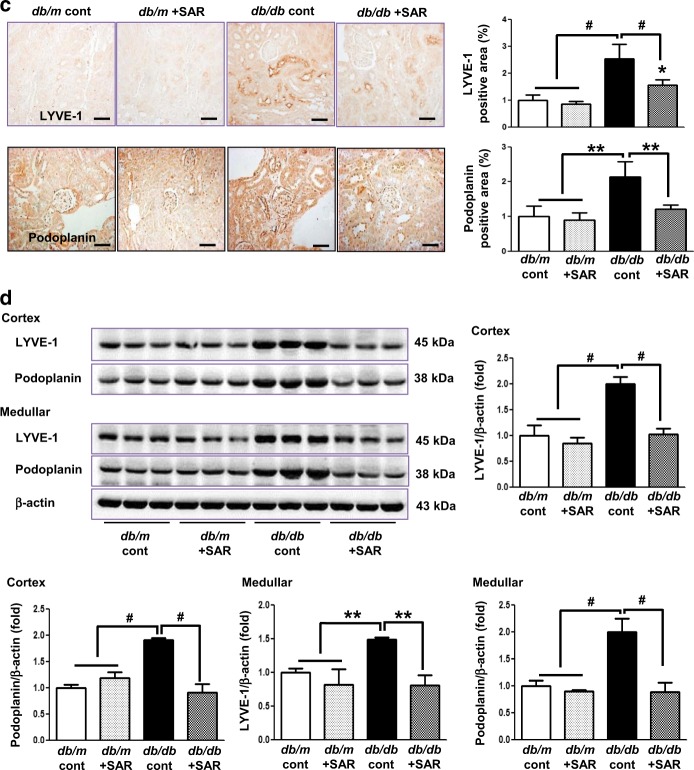
SAR131675 attenuates transforming growth factor (TGF-β) expression and lymphangiogenesis in the kidneys in *db/db* mice. Effects of SAR131675 on the expression of TGF-β, vascular endothelial cell growth factor-C (VEGF-C), vascular endothelial cell growth factor receptor-1 (VEGFR-1), VEGFR-2, VEGFR-3, LYVE-1, and podoplanin in the cortex and medulla were determined at 20 weeks in *db/m* and *db/db* mice treated with or without SAR131675. **a** Representative immunohistochemistry for TGF-β and VEGF-C. **b** Representative western blots for VEGF-C, VEGFR-1, VEGFR-2, VEGFR-3, and β-actin in the cortex and medullar and quantitative data. **c** Representative images of immunohistochemical staining for LYVE-1 and podoplanin, and **d** western blots for LYVE-1, podoplanin, and β-actin in the cortex and medullar and quantitative data. *P *<* 0.05, **P *<* 0.01, and ^#^*P* < 0.001 vs. other groups

Interestingly, the expression of TGF-β, TNF-α, VEGF-C, and VEGFR-3 in the cortex and medulla of the kidneys of *db/db* mice increased (Figs 2c, 3a, b). Consistently, LYVE-1 and podoplanin, which are lymphovascular endothelial cell markers, significantly increased in the *db/db* control group as indicated by immunohistochemistry and western blotting (Fig. 3c, d). TGF-β- and TNF-α-induced lymphangiogenesis in *db/db* mice was markedly reversed to the level of *db/m* mice upon SAR131675 treatment (Fig. 3a–d). In contrast, SAR131675 treatment did not affect VEGFR-1 and VEGFR-2 expression levels in *db/db* mice. These results suggested that SAR131675 selectively affects TGF-β- and TNF-α-induced VEGF-C-VEGFR-3 expression and subsequently inhibits intrarenal lymphangiogenesis in *db/db* mice.

### Effects of SAR131675 treatment on glomerular and tubulointerstitial phenotypes

**Fig. 4:**
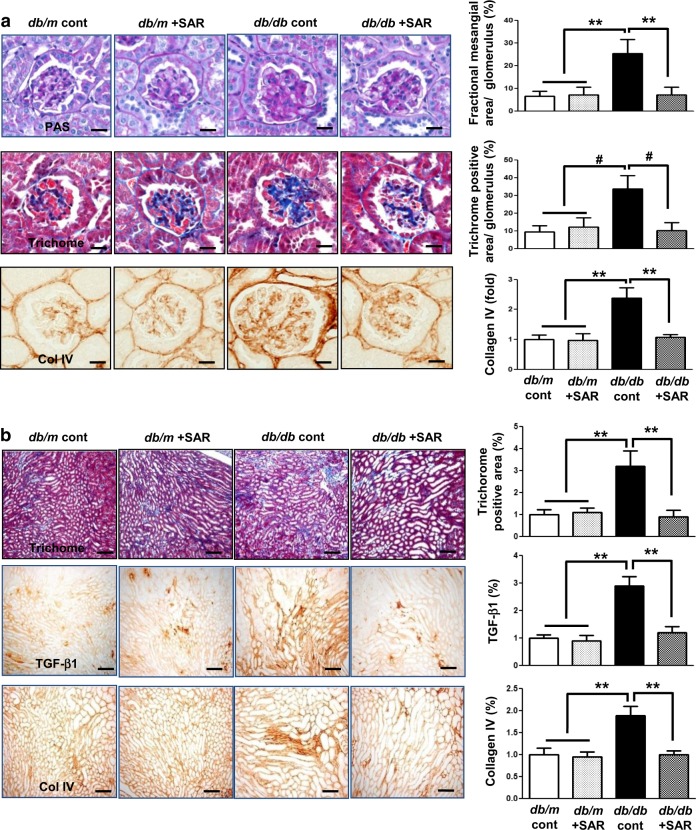

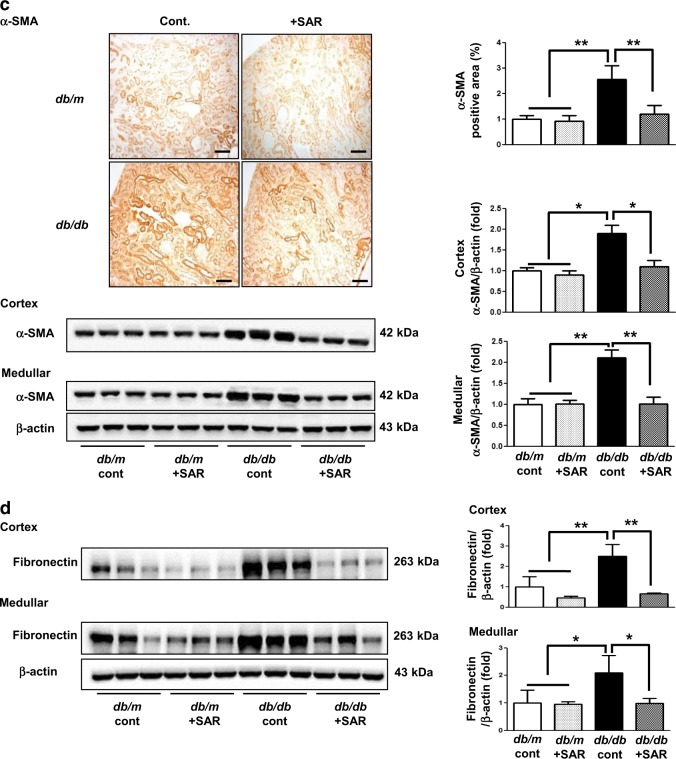
SAR131675 decreases glomerulosclerosis and tubulointerstitial fibrosis in *db/db* mice. Effects of SAR131675 on glomerulosclerosis and tubulointerstitial fibrosis were determined at 20 weeks in *db/m* and *db/db* mice treated with or without SAR131675. **a** Representative images of periodic acid Schiff (PAS) and trichrome staining and immunohistochemistry for type IV collagen (Col IV) and quantitative data. **b** Representative images of trichrome staining and immunohistochemistry for transforming growth factor (TGF-β) and Col IV and quantitative data. **c** Representative images of immunohistochemistry for α-smooth muscle actin (α-SMA) and western blots for α-SMA and β-actin in the cortex and medullar and quantitative data. **d** Representative western blots for fibronectin and β-actin in the cortex and medullar and quantitative data. *P < 0.05, **P < 0.01, and ^#^*P* < 0.001 vs. other groups

Increased extent of glomerulosclerosis and tubulointerstitial fibrosis in *db/db* mice as indicated by increased expression of Col IV, TGF-β1, α-SMA, fibronectin, and Masson’s trichrome-positive areas were suppressed by SAR131675 treatment (Fig. 4a–d).

### Effects of SAR131675 treatment on glomerular apoptosis and oxidative stress in the kidneys

**Fig. 5:**
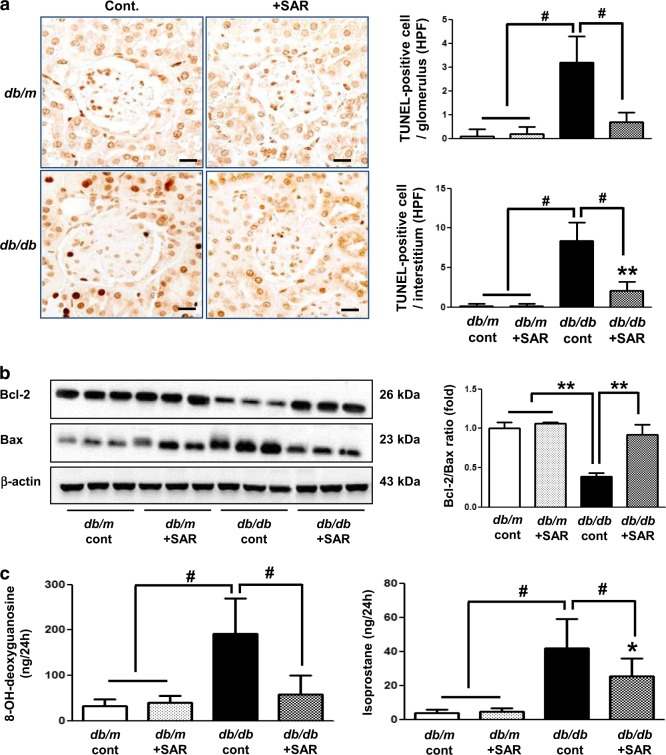
SAR131675 attenuates apoptosis and oxidative stress in the kidneys in *db/db* mice. Glomerulosclerosis and tubulointerstitial fibrosis were determined at 20 weeks in *db/m* and *db/db* mice treated with or without SAR131675. **a** Representative images of terminal deoxynucleotidyl transferase-mediated dUTP nick-end labeling (TUNEL) staining in the glomeruli and tubules and quantitative data. **b** Representative western blots for B cell leukemia/lymphoma 2 (Bcl-2), Bcl-2-associated X protein (Bax), and β-actin in the kidneys and quantitative data. **c** Twenty-four hour urinary 8-hydroxy-deoxyguanosine (8-OH-dG) and isoprostane levels. *P < 0.05, **P < 0.01, and ^#^*P* < 0.001 vs. other groups

Inflammatory cell infiltration is known to cause cellular damage and oxidative stress in the kidneys, leading to a vicious cycle of inflammation and apoptosis. In the current study, *db/db* mice demonstrated increased number of TUNEL-positive cells and decreased expression of Bcl-2/Bax ratio and corresponding increases in renal oxidative stress markers including urinary 8-OH-dG and isoprostane (Fig. 5). Importantly, these diabetic alterations were reduced upon SAR131675 treatment, suggesting its favorable role in ameliorating apoptotic cell death and oxidative stress in diabetic kidneys.

### Effects of SAR131675 treatment on HK2 and RAW264.7 cells

**Fig. 6:**
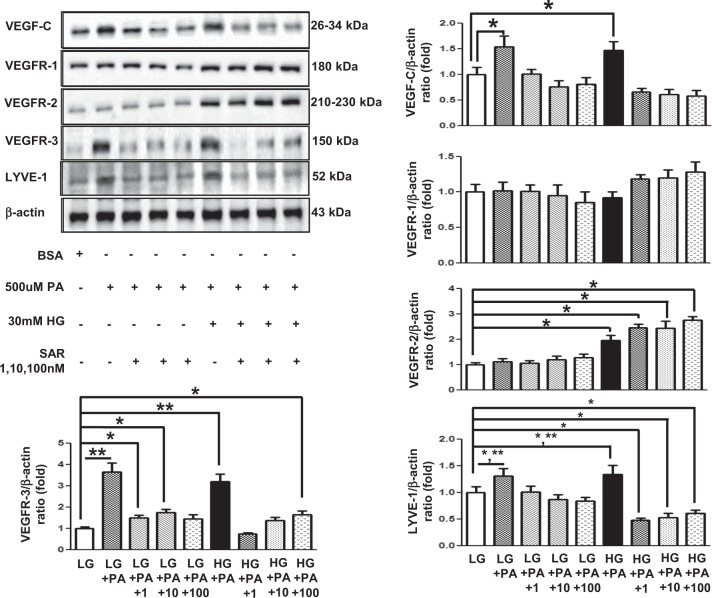
SAR131675 decreases high-glucose and palmitate-induced vascular endothelial cell growth factor-C (VEGF-C), vascular endothelial cell growth factor receptor-3 (VEGFR-3), and LYVE-1 expression in human kidney-2 (HK2) cells. To determine whether the addition of SAR131675 might modulate lymphangiogenesis in human proximal tubular epithelial HK2 cells, the cells were stimulated with palmitate (500 μM) and exposed to SAR131675 at 1, 10, or 100 nM in low-glucose (LG; 5 mmol/L d-glucose) or high-glucose (HG; 30 mmol/L d-glucose) medium. Representative western blots for VEGF-C, VEGFR-1, VEGFR-2, VEGFR-3, and β-actin and quantitative data. *P *<* 0.05, **P *<* 0.01 vs. LG control

**Fig. 7:**
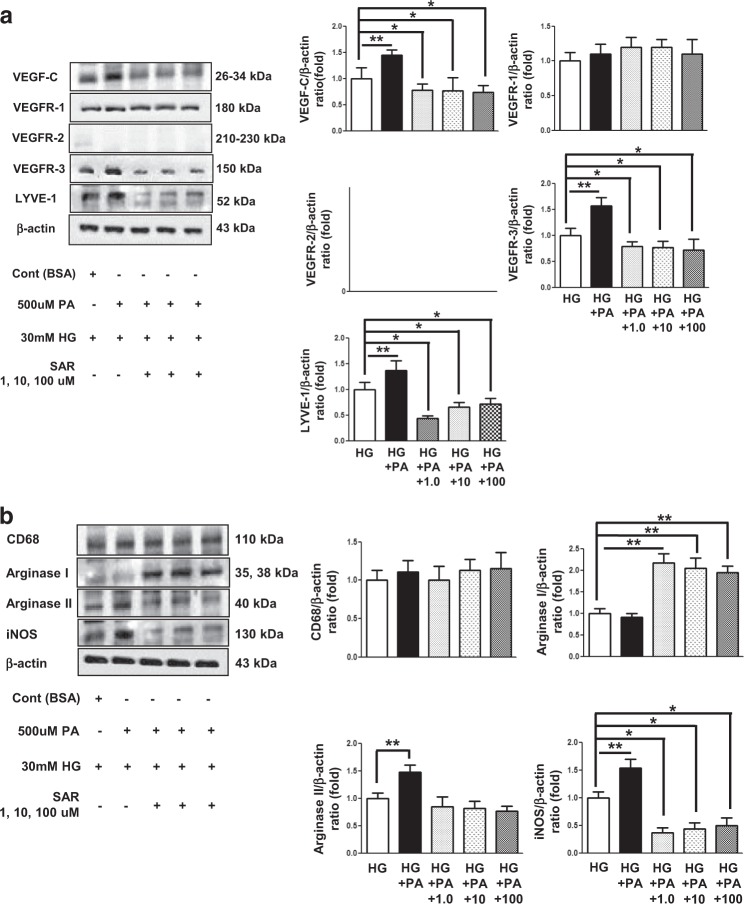

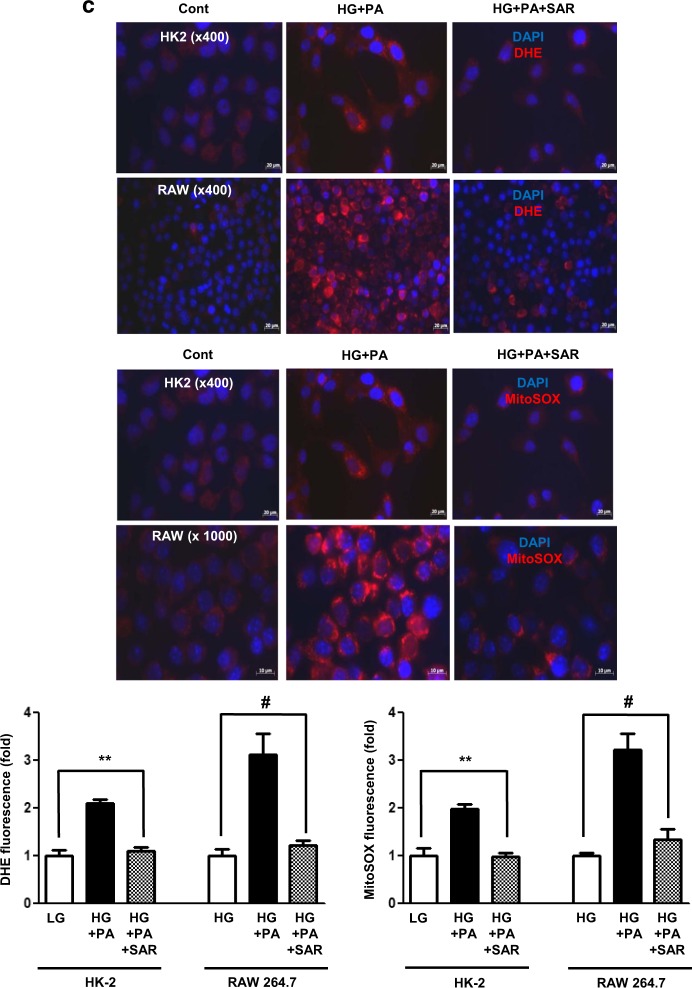
SAR131675 decreases high-glucose and palmitate-induced vascular endothelial cell growth factor-C (VEGF-C), vascular endothelial cell growth factor receptor-3 (VEGFR-3), and LYVE-1 expression and M polarization in RAW264.7 cells. To determine whether SAR131675 might modulate lymphangiogenesis and macrophage polarization in RAW264.7 murine macrophage cells, the cells were stimulated with palmitate (500 μM) in high-glucose medium (HG; 30 mmol/l d-glucose) and exposed to SAR131675 at 1, 10, and 100 nM. **a** Representative western blots for VEGF-C, VEGFR-1, VEGFR-2, VEGFR-3, and β-actin and quantitative data. **b** Representative western blots for CD68, arginase I, and arginase II, inducible nitric oxide synthase (iNOS), and β-actin and quantitative data. **c** Representative confocal microscopy images showing dihydroethidium (DHE) and MitoSOX fluorescence in the human kidney-2 (HK2) and RAW264.7 cells and quantitative data. *P < 0.05, **P < 0.01, ^#^*P* < 0.001 vs. control

We analyzed the therapeutic effect of SAR131675 on palmitate-induced lipotoxicity in human proximal tubular epithelial HK2 cells and murine RAW264.7 macrophages, which play a major role in lymphangiogenesis in the kidneys (Figs 6 and 7). In both HK2 cells and RAW264.7 cells, the expression of VEGF-C, VEGFR-3, and LYVE-1 was significantly increased following palmitate treatment, whereas VEGFR-1 and VEGFR-2 did not change (Figs 6a and 7a). M1/M2 macrophage polarization plays a crucial role in lymphangiogenesis. Therefore, we evaluated macrophage polarization after palmitate-induced stress by distinguishing macrophage phenotypes. Arginase II and iNOS, which are M1 macrophage markers, were significantly increased in RAW246.7 cells after palmitate treatment (Fig. 7b), whereas no changes were observed in the expression of CD68, and arginase I, which denotes M2 macrophage phenotype. These findings suggested that palmitate-induced lipotoxicity favors M1 polarization, in accordance with lymphangiogenesis. Importantly, SAR131675 effectively prevented palmitate-induced increase in the expression of VEGF-C, VEGFR-E, and LYVE-1 in HK2 and RAW264.7 cells (Figs 6 and 7) by inhibiting M1 polarization in RAW264.7 cells (Fig. 7b). Additionally, in response to palmitate-induced oxidative stress, superoxide anion (O_2_^•−^) levels measured by DHE and MitoSOX fluorescence were attenuated in SAR131675 (10 nM)-treated group (Fig. 7c).

## Discussion

The current study showed that SAR131675 treatment lowered systemic dyslipidemia and the levels of inflammatory cytokines, and ameliorated lipotoxicity-induced inflammation—as reflected by reduced MCP-1 and TNF-α expression and M1 macrophage infiltration and oxidative stress in the kidneys, thus suppressing glomerulosclerosis, tubulointerstitial fibrosis, and apoptotic renal cell death. Interestingly, SAR131675 treatment significantly decreased diabetes-induced increase in the expression of VEGFR-3 and VEGF-C in the whole kidneys, suggesting a suppression of lymphangiogenesis, which was supported by decreases in the expression of lymphatic endothelial markers LYVE-1 and podoplanin that were further associated with the prevention of glomerulosclerosis and tubulointerstitial fibrosis. In experiments with HK2 and RAW264.7 cells, palmitate-induced increase in the expression of VEGF-C and VEGFR-3 and accentuation of M1 polarization, along with exacerbation in cytosolic and mitochondrial oxidative stress, were inhibited by SAR131675 treatment. Taken together, these results indicated that SAR131675 might prevent lipotoxicity-induced lymphangiogenesis in type 2 diabetes.

One of the prime factors that contributes to the pathogenesis of diabetic nephropathy is chronic hyperglycemia. Continuous hyperglycemia causes systemic dyslipidemia and increases free fatty acid synthesis and triglyceride accumulation (lipotoxicity) in adipose tissue and other parenchymal organs including the liver, the heart, and the kidneys. In the kidneys, excessive lipid accumulation stimulates TGF-β and TNF-α expression and ROS production, which induce an inflammatory response, glomerulosclerosis, and tubulointerstitial fibrosis, leading to diabetic renal damage^17^. Our results showed that SAR131675 treatment significantly reduced dyslipidemia and intrarenal lipid accumulation to the levels comparable to that of non-diabetic *db/m* mice and subsequently decreased TGF-β and TNF-α levels and oxidative stress.

Lymphangiogenesis occurs in both physiologic and pathologic conditions. In general, lymphangiogenesis occurs at sites of tissue inflammation, suggesting the existence of a mutual interaction between inflammation and lymphangiogenesis^18^. The most common feature of macrophages in both lymphangiogenesis and angiogenesis is that they secrete paracrine signaling molecules. While VEGF-C is known to stimulate angiogenesis, it is often associated with lymphangiogenesis as well. In other words, VEGF-C is the most important factor in lymphangiogenesis^19^. Suzuki et al.^20^ suggested that TGF-β induces upregulation of VEGF-C expression in macrophages, leading to lymphangiogenesis. Another main pathway for lymphangiogenesis is a TNF-α-dependent signaling pathway, which is entirely dependent on VEGFR-3 activation^21^. Moreover, recruited macrophages can be derived from monocytes that are activated in the bone marrow by chemokines such as MCP-1 and enter the bloodstream and traffic to sites of tissue injury. In the current study, the increased systemic and renal MCP-1 and TNF-α expression in *db/db* mice might have originated from recruited intrarenal macrophages that polarized to M1 and subsequently increased TGF-β- and TNF-α-dependent lymphangiogenesis, which ultimately led to glomerulosclerosis and tubulointerstitial fibrosis. Interestingly, SAR131675 treatment improved pro-inflammatory lymphangiogenesis and reduced diabetic renal damage.

During embryonic development, the maintenance of strong VEGFR-3 expression in lymphatic endothelial cells together with reduced VEGFR-3 expression in vascular endothelial cells probably indicates that these cells are committed to lymphangiogenesis^22^. In an experimental autoimmune orchitis model used to study chronic inflammation of the testes, F4/80 macrophages were found to migrate to and be incorporated into the lymphatic capillary walls, but not the walls of blood capillaries^21,23^. Similarly, El-Chemaly et al.^24^ reported that CD11b+ macrophages formed lymphatic-like structures in Matrigel in vitro that were positive for LYVE-1 and podoplanin. These results indicate that these markers are associated with pro-inflammatory lymphangiogenesis. The current study clearly showed that increased F4/80 and M1-polarized macrophages were associated with VEGF-C- and VEGFR-3-dependent lymphangiogenesis.

In keeping with an expanding body of evidence that resident and infiltrating macrophages and proximal tubular epithelial cells are major contributors to the pathogenesis of lymphangiogenesis-associated renal damage, we explored changes in relevant signaling in both HK2 and RAW264.7 cells. VEGF-C, VEGFR-3, and LYVE-1 expression was increased upon exposure to palmitate in HK2 cells, but was normalized to the levels comparable to that of low glucose without palmitate after SAR131675 treatment, whereas the expression of VEGFR-1 and VEGFR-2 was not altered by SAR131675 treatment. In RAW264.7 macrophages, the changes in the expression of VEGFR-3 before and after the treatment were similar upon exposure to palmitate.

It was recently reported that polymorphism of (pro-inflammatory) M1 and (anti-inflammatory) M2 macrophages plays an important role in the inflammatory response^25^. The M2 phenotype is classified into M2a, b, c, and d. M2b is characterized by immune complex formation and Toll-like receptor activation. M2c represents deactivated macrophages that suppress pro-inflammatory cytokines, and M2d are regulatory macrophages that are often grouped with tumor-associated macrophages (TAMs)^12,26^. TAMs express VEGFR-3 on the membrane surface and express VEGF-C or VEGF-D following TNF-α stimulation, and they contribute to the formation of lymphatic vessels by direct transdifferentiation into lymphatic endothelial cells^27,28^. Recently, the generalization of TAMs as M2-like alternatively activated cells has been challenged by new evidence that a spectrum of macrophages exists in tumors, and the M2/M1 macrophage ratio may in fact be more predictive of a poor prognosis than total infiltration^21,29^. Current evidence suggests that these populations may vary based on their localization within the tumor, with pro-angiogenic M2-like macrophages congregating in hypoxic areas and pro-inflammatory M1 macrophages congregating in regions of tissue inflammation^30^. In our aim to study the inflammatory cells in more detail, we found that infiltrating macrophages in the kidneys were of the M1 phenotype. In RAW264.7 cells, palmitate-induced lipotoxicity favored M1 polarization, but not M2 polarization, and this was responsible for pro-inflammatory lymphangiogenesis, as indicated by increases in the expression of VEGF-C, VEGFR-3, and LYVE-1.

It has been known that enhanced formation of ROS and accelerated lipid peroxidation processes have been detected in lymphoedematous tissue, which are considered to contribute to the damage of lymphatic endothelium^31,32^. The expression of VEGFR-3 in lymphatics, along with cellular anti-oxidative defense molecules, likely plays a major role in protecting against oxidative stress-induced cell damage. The functional loss of VEGFR-3 facilitates disease-related pathological processes, which is associated with failing to activate Akt and to prevent cell death under oxidative stress^33^. Therefore, the current study (both in vivo and in vitro studies performed in RAW264.7 and HK2 cells) demonstrated that the inhibition of VEGFR-3 by SAR131675 might ameliorate lipotoxicity-induced oxidative stress in diabetic kidney through inhibition of dysfunctional lymphangiogenesis.

In conclusion, the current study demonstrated evidences that diabetes-induced intrarenal lipotoxicity and subsequent macrophage infiltration were accompanied by lymphatic proliferation that was in parallel with the extent of renal fibrosis, oxidative stress, and apoptosis. In this process, removal of intrarenal inflammatory cells and toxic lipid metabolites may have been facilitated through restoration of functional lymphatics by SAR13175 treatment, resulting in marked functional and phenotypic improvement in the kidney. Therefore, SAR111675 may attenuate inflammatory response, oxidative stress, and apoptotic cell death through restoration of functioning lymphatics, which ultimately ameliorates renal fibrosis in type 2 diabetes-induced renal lipotoxicity. Our findings provide a better understanding of the role of lymphatics in the pathogenesis of diabetic nephropathy and pave the way to exploring new therapies for the inveterate disease, diabetic nephropathy.
